# Belladonna in Broncho-Pneumonia in Children

**Published:** 1899-02-04

**Authors:** 


[1] Brit. Med. Jour., Jan. 28.

### BELLADONNA IN BRONCHO-PNEUMONIA IN CHILDREN.

Dr. Coutts, physician to the Shadwell Children's Hospital, draws attention[1] to the good results which in his hands have followed the employment of belladonna in the treatment of the broncho-pneumonia of children. Its utility in that very fatal sequela of diphtheria—paralysis of the diaphragm—has been known for some time, although it was difficult to find any satisfactory explanation of its action. It was, however, pointed out some time ago by Dr. Ringer that atropine had a marked effect in limiting or diminishing the secretion from the bronchial tubes and pulmonary tissues, and it seemed to Dr. Coutts that this might not only be the explanation of its good effect in these cases of paralysis of the diaphragm (by preventing the secondary pulmonary consequence of the paralysis), but that it suggested a possible field of usefulness for the drug in the broncho-pneumonia of children, cases in which the little patients are suffocated by the excessive secretion with which their bronchial and pulmonary tissues are filled. "So far," he says, "it has more than answered all I could have hoped for. With it, as the sole drug administered, there has been in my cases no need for steam tents, oxygen inhalations, unlimited stimulations, and all the rest of the former varied and trying treatment." The preparation used has been the extract of the late Pharmacopœia. "This extract I have given in doses of ¼ grain every three or four hours. I have made no distinction, too, in the dose as regards the age of the patient, and have given the same dose to an infant a few weeks old as to a child of six or seven years."

